# A Zebrafish Seizure Model of cblX Syndrome Reveals a Dose-Dependent Response to mTor Inhibition

**DOI:** 10.3390/jdb14010002

**Published:** 2025-12-25

**Authors:** Claudia B. Gil, David Paz, Briana E. Pinales, Victoria L. Castro, Claire E. Perucho, Annalise Gonzales, Giulio Francia, Sepiso K. Masenga, Antentor Hinton, Anita M. Quintana

**Affiliations:** 1 Department of Biology, University of Texas Arlington, 701 S Nedderman Dr., Arlington, TX 76019, USA; claudia.gil@uta.edu; 2 Department of Biological Sciences, The University of Texas El Paso, 500 West University Ave, El Paso, TX 79968, USA; dpaz3@utep.edu (D.P.); bepinales@utep.edu (B.E.P.); ciperucho@miners.utep.edu (C.E.P.); avgonzales@miners.utep.edu (A.G.); gfrancia@utep.edu (G.F.); 3 Department of Developmental Neurobiology, St. Jude Children’s Research Hospital, 262 Danny Thomas Dr., Memphis, TN 38105, USA; victoria.castro@stjude.org; 4 Department of Molecular Physiology and Biophysics, Vanderbilt University, Nashville, TN 37232, USA; sepisomasenga@lcpts.org (S.K.M.);

**Keywords:** cblX, HCFC1, intractable/focal epilepsy, mTor

## Abstract

Mutations in the transcriptional co-factor HCFC1 cause methylmalonic aciduria and homocystinemia, cblX type (*cblX*) (MIM#309541), non-syndromic X-linked intellectual disability (XLID), and focal epilepsy. Zebrafish studies have revealed increased activation of the Akt/mTor signaling pathway after mutation of *hcfc1a*, one ortholog of *HCFC1*. mTOR hyperactivation is linked to seizures, and its inhibition alleviates epilepsy in other preclinical models. We hypothesized that mTor overactivity in *hcfc1a* mutant zebrafish increases seizure susceptibility and/or severity. We employed a two-concentration model of the seizure-inducing agent, pentylenetetrazol (PTZ), with or without pretreatment of the mTor inhibitor, torin1. Mutation of *hcfc1a* did not alter the response to PTZ at sub-optimal concentrations, and the pharmaceutical inhibition of mTor using the compound Torin1 reduced response to 1 µM PTZ, but only in a dose-dependent manner. Higher doses of mTor inhibition did not reduce the seizure response in mutant larvae but were effective in wildtype siblings. These data suggest that inhibition of mTor in an *hcfc1a*-deficient background leads to a reaction that differs from the traditional response observed in wildtype siblings. Collectively, we present a model that can be used to test dose–response and the development of combinatorial treatment approaches in a high-throughput manner.

## 1. Introduction

*HCFC1* encodes a multi-domain transcriptional co-factor that regulates the expression of more than 5000 genes [1]. Mutations in the kelch protein interaction domain of HCFC1 cause methylmalonic aciduria with homocystinemia, type cblX (*cblX*), a multiple congenital anomaly syndrome characterized by abnormal vitamin B12 metabolism, craniofacial dysmorphia, neurodevelopmental defects, failure to thrive, and intractable epilepsy [2]. Mutations in other domains of HCFC1 cause non-syndromic X-linked intellectual disability (XLID) or focal epilepsy [3,4,5,6,7]. These disorders highlight the function of HCFC1 in neural development and indicate that dysregulation of HCFC1 can cause seizures or intractable epilepsy. The mechanisms underlying seizure phenotypes are currently unknown. However, various functional studies across multiple model systems indicate that HCFC1 regulates neural development and a key developmental pathway, Akt/mTor, which has been implicated in seizure phenotypes.

Functional analysis has demonstrated that HCFC1 is required to maintain the number and proliferation of neural precursor cells (NPCs) and neurite outgrowth [3,4]. Subsequent studies in zebrafish validated the increased number and proliferation of NPCs through morpholino-mediated knockdown of *hcfc1b* and germline mutation of the *hcfc1a*, the two zebrafish orthologs of *HCFC1* [8,9]. Nonsense mutation of murine *HCfC1* in a sub-population of NPCs resulted in increased cell death and reduced GABAergic cells [10], the primary inhibitory cells of the central nervous system, whose dysfunction has been implicated in epilepsy and seizures. A genetic knock-in allele of *cblX* syndrome was created in mice, but brain phenotypes were not comprehensively studied [11]. Nonsense mutation of the zebrafish *hcfc1a* gene (*hcfc1a^co60/+^*) results in increased expression of the *asxl1* gene [9]. *Asxl1* encodes a polycomb repressor protein with functions in the nucleus and cytoplasm. The role of ASXL1 in the cytoplasm leads to hyperphosphorylation of AKT kinase in mouse embryonic fibroblasts [12]. AKT kinase regulates cell growth, proliferation, and survival.

The function of HCFC1 in the indirect regulation of AKT activity is the only current mechanism that can be linked to seizure phenotypes in *cblX* syndrome. In humans, mutations in the AKT pathway are associated with epileptic phenotypes [13]. Interestingly, inhibition of Akt (zebrafish) phosphorylation and activation restores NPC overproduction in the *hcfc1a^co60/+^* haploinsufficient zebrafish, providing evidence that Akt and its downstream pathways underlie some neurodevelopmental phenotypes observed in the mutant larvae. Subsequent experiments demonstrated hyperactivation of mTor (mammalian target of rapamycin), a downstream target of Akt, in the *hcfc1a^co60/+^* zebrafish [14]. These data raise the possibility that hyperactivation of mTOR could underlie seizure phenotypes in *cblX*. This is because hyperactivation of AKT/mTOR signaling has been extensively shown to underlie major seizure disorders, including tuberous sclerosis complex [15,16,17,18]. Based on these data, we hypothesized that mutation of *hcfc1a* leads to increased seizure susceptibility and severity due to hyperactivation of mTor.

In utero seizures have been detected in humans with mutations in *HCFC1* [19]. Thus, we opted to monitor seizure phenotypes in larval zebrafish after exposure to pentylenetetrazol, a GABA receptor antagonist established to cause seizures in multiple model systems [20,21,22,23,24,25,26]. We selected the *hcfc1a^co60/+^* genotype for our studies because these zebrafish carry a nonsense mutation in the kelch domain, are heterozygous viable, and have increased numbers of NPCs with hyperactive mTor signaling [9,14]. We exposed larval zebrafish (*hcfc1a^co60/+^* offspring) to established seizure-inducing concentrations of PTZ and sub-optimal doses (a dose that has no behavioral effect on wildtype animals). The sub-optimal dose did not increase sensitivity to PTZ in larvae with the *hcfc1a^co60/+^* allele. Wildtype siblings had a reduced response to PTZ when pretreated with mTor inhibitors, and the efficacy of pharmaceutical inhibition improved with higher concentrations of mTor inhibition. In contrast, the efficacy of pretreatment in mutant larvae was dose-dependent, with lower concentrations showing improved efficacy and higher concentrations exacerbating the seizure-like phenotypes according to the metrics evaluated. A substantial amount of mTor remained active in mutant larvae despite pretreatment with an mTor inhibitor, substantiating dysregulation of mTor in mutants. These data indicate that while mutation of *hcfc1a* and hyperactivation of mTor do not increase seizure susceptibility, the manipulation of mTor, in the context of *hcfc1a* mutation, can contribute to intervention efficacy and can promote a deleterious response at high doses, which show improved efficacy in wildtype animals. Follow-up combinatorial therapies with a full panel of inhibitors, multiple treatment windows, and additional dose–response curves are warranted. These follow-up studies can be tested in a high-throughput manner when used in combination with our current model system and seizure paradigm.

## 2. Materials and Methods

### 2.1. Zebrafish and Genotyping

For the following experiments, embryos are produced from natural spawning of the following lines: *hcfc1a^co60/+^*, AB, Tupfel long fin, or AB crossed with Tupfel long fin. The *hcfc1a^co60/+^* is maintained in a background that carries AB and Tupfel long fin genetic inheritance. Embryos are maintained in E3 media (embryo medium) at 28 °C with a 14/10 light: dark cycle. All procedures were approved by the Institutional Animal Care and Use Committee at the University of Texas at El Paso, protocol number 811869-5. Methods for euthanasia and anesthesia were performed according to guidelines from the 2020 American Veterinary Medical Association guidebook.

All adults and larvae were genotyped using polymerase chain reaction (PCR). Tissue was lysed in 50 millimolar (mM) sodium hydroxide (Fisher Scientific, Waltham, MA, USA) for 10 min at 95 °C and pH-adjusted with 500 millimolar (mM) Tris-HCl. Allele-specific primer pairs were used for each allele. These primers bind to and amplify the mutated allele but do not amplify the wildtype allele (5). For the *hcfc1a*^co60/+^ allele, the primers specific to the mutated allele were FWD: CCAGTTCGCCTTTTTGTTGT and REV: ACGGGTGGTATGAACCACTGGC, each used at a final concentration of 0.5 mM. PCR annealing was executed at 64 °C and 30–35 cycles were performed. GoTaq Green (1X) was used for all genotyping (Fisher Scientific, Waltham, MA, USA).

### 2.2. Reagents

We performed genotyping using GoTaq green (Promega, Madison, WI, USA) and primers from Millipore Sigma, St. Louis, MO, USA, synthesized at the 0.025 µmole scale with desalt purification. Primers are resuspended at 100 µM stock concentration in purified water. E3 media is produced according to the previously described recipe [27], and all reagents were purchased from Fisher Scientific Waltham, MA, USA (NaCl, KCl, CaCl_2_ * 2H_2_O, MgCl_2_ * 6H_2_O). Torin1 was purchased from Selleck chemicals Houston, TX, USA, catalog # S2827, with 99.07% purity. PTZ was purchased from Millipore Sigma St. Louis, MO, USA (Catalog #P6500) in a 25G size. Dimethyl sulfoxide (DMSO) was purchased from Fisher Scientific Waltham, MA, USA, catalog # J666501.AE. Antibodies for Western blot were purchased from Cell Signaling Danvers, MA, USA (pS6 catalog # 4858S) or Millipore Sigma St. Louis, MO, USA (actin catalog # A5441).

### 2.3. Zebrabox Behavioral Analysis

Behavioral analysis was performed using the Zebrabox technology (ViewPoint Behavioral Technology, Montreal, QC, Canada). The Zebrabox is a self-contained unit with built-in controls. The analysis performed here was performed in dark conditions with an infrared backlight used for video tracking. This is controlled by the unit. For temperature control, the Zebrabox was placed in a temperature-controlled room. All analysis is performed as previously optimized [24,25,26], between peak hours of activity, as previously determined [28], and the arena size [29] is consistent across biological replicates (48-well dish with 1.5 mL of E3 total volume). Analysis was performed at 5 days post-fertilization (dpf). The presence of a swim bladder was confirmed prior to the analysis. Larvae were individually placed into a 48-well plate with E3 media. All larvae were acclimated in dark conditions for 1 h before data collection. The behavioral assay lasted for a total duration of 10 min in the dark. After acclimation, behavior was recorded for 5 min in the dark to obtain a baseline measurement (no PTZ). Parameters were developed based on previous studies [30,31], except for black detection being used instead of the transparent setting, and the threshold was set to 25 to provide improved detection in dark conditions. The Zebrabox reports the duration (s) and distance of swimming (mm). Swimming measurements are split into small (4–20 mm/s) and large/fast (20 mm/s). The parameters, total distance, large distance, small distance, speed, large duration, small duration, large count, and small count, were analyzed to determine changes in locomotor activity and seizure-like behavior. The raw data was recorded on an Excel sheet along with a video file. Speed and total distance were manually calculated as described [30]. Total distance is the sum of large and small distances, and speed is calculated by dividing total distance by the sum of small and large duration parameters (mm/s).

### 2.4. Torin1 and PTZ Inhibition

A sub-optimal concentration of PTZ was derived empirically by performing a concentration gradient and monitoring seizure-like behaviors (locomotion, distance, time, and speed). The gradient was performed using wildtype larvae at 5 dpf. For each concentration, a total of 48 animals were evaluated. The total number of larvae was obtained over the course of 3 biological replicates. Each biological replicate was derived from different parents on different days, and a collective analysis with a total of 48 animals per group was evaluated and is indicated in the radar plots shown. For behavioral analysis, larvae were individually placed into a 48-well plate with 1450 µL of E3 media. After acclimation and a 5 min baseline recording, PTZ was added to the following final concentrations: 1 µM, 10 pM, 1 pM, 0.1 pM, 0.01 pM, and 0.001 pM. Behavior was recorded for 5 min in the dark after exposure to PTZ. To test the effects of the sub-optimal dose on mutants and their sibling clutchmates, a total of 92 larvae were blindly evaluated. The 92 larvae were obtained from different crosses on different days over the course of 3 replicates. The Figures show representative animals from each biological replicate (Rep1, Rep2, and Rep3). The *hcfc1a^co60/+^* allele does not obey Mendelian inheritance ratios, due to the absence of homozygous animals, thus requiring more larvae in some cases to obtain enough animals for statistical significance. Larvae that did not move under baseline conditions were excluded from the analysis. From the larger clutch, a total of 42 larvae were included in the final analysis. Some larvae were not included due to an experimental error associated with genotyping. There were a total of 23 *hcfc1a*^co60/+^ larvae and 19 siblings. For treatment assays, within each biological replicate, larvae from a large pool of clutches were pooled and split to include all treatment groups and internal controls. Representative wells from each biological replicate, performed on different days, are shown in the Figures. The total number of larvae analyzed was 145 across all replicates. For each group/clutch, stage, growth conditions, and water quality were controlled. All behavioral analysis was blinded and performed prior to genotyping. Animals without a baseline movement were excluded. Less than 0.1% of animals were excluded, and variability was included in the bar graphs as standard error of the mean. Trace patterns for each biological replicate are indicated in the Figures (Rep1, Rep2, and Rep3).

For treatment with vehicle control, the concentration of DMSO for each group was never more than 0.01%. A comparison between 0.01% and 0.1% was performed in our initial optimization of torin1 in the Figures. A concentration of 0.01% was selected for all subsequent experiments. Previous studies indicate that concentrations of DMSO up to 1% are safe for zebrafish larvae, and the most recommended concentration is 0.01% [32]. However, these previous studies do not specifically test for pS6 and only identify lethality and malformations. Additional studies using 0.1–1% DMSO have evaluated larval behavior, and concentrations > 0.55% alter behavior [33]; however, we used much less, and no behavioral deficits appeared in our controls (Figure S2). Our data are supported by previous studies in 5-day-old larvae across multiple behavioral tests [34]. The following parameters, total distance, large distance, small distance, speed, large duration, small duration, large count, and small count, were analyzed to determine changes in locomotor activity and seizure-like behavior in response to the drug treatment. Seizure-like behavior was interpreted using the zebrafish behavioral catalog [35].

Torin1 is a selective inhibitor of mTOR (both complex 1 and 2), causing reduced cell growth and proliferation [36]. Western blot analysis was performed, as previously described [14], to determine the appropriate concentration of torin1 required to reduce the level of phosphorylated S6 ribosomal protein in wildtype larvae. For all analyses shown here, we show a single biological replicate using a pooled sample. This is provided as a representative example; however, all Western blots were performed in biological duplicate with a minimum of 14 embryos per pool. The biological replicates for validatory data with torin1 treatmentis also provided in Supplemental Data (Figure S3), and a collation of normalized density from both replicates is shown in the Figures. Based on these data, a concentration of 250 nM of torin1 was deemed the minimal concentration to reduce phosphorylated S6 ribosomal protein, without resulting in abnormal development (gross morphological defects). Torin1 treatment was initiated at 24 h post-fertilization (hpf) to allow for early development. Torin1 was replaced daily for 4 days prior to behavioral analysis, and treated larvae were challenged with PTZ at 5 dpf. To assess the effects of torin1 treatment on seizure severity, 1 µM PTZ was used to induce seizure-like behavior.

### 2.5. Statistical Analysis

An ANOVA was used to determine the statistical differences between multiple groups, followed by a post hoc *t*-test between individual groups. All data reported statistically significant has a *p*-value < 0.05. In all cases, a paired *t*-test was used to compare no PTZ treatment with PTZ treatment, because the same animals were used for baseline and PTZ measurement.

## 3. Results

### 3.1. Larval Response to Low-Dose PTZ

**Figure 1:**
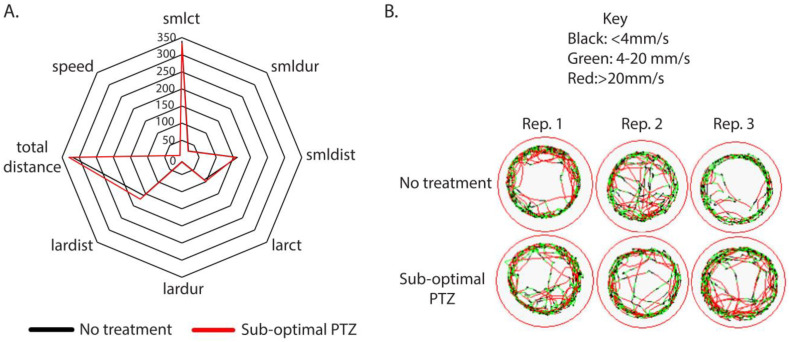
Empirical derivation of a sub-optimal concentration of PTZ. (**A**,**B**) Wildtype larvae (5 days post-fertilization) were individually placed in a 48-well dish and acclimated for 1 h at room temperature to the ZebraBox self-contained environment. Individual larvae were monitored for 5 min in dark conditions to obtain a baseline behavior. After recording the baseline behavior, larvae were exposed to pentylenetetrazol (0.001 pM). A representative radar plot was used to analyze all parameters simultaneously. (**A**). Black lines in the radar plot represent animals that were untreated (no PTZ), and red lines indicate animals that were treated with 0.001 pM PTZ. Representative trace patterns are shown (**B**). Abbreviations: Rep: Replicate. Representative trace patterns are from 3 independent biological replicates. The total number of animals (48) was obtained from 3 independent replicates.

We hypothesized that nonsense mutation of *hcfc1a* causes increased seizure susceptibility due to an underlying hyperactivation of mTor signaling [14]. To test this hypothesis, we first sought to identify a sub-optimal dose of the seizure-inducing agent PTZ by performing a concentration gradient on wildtype larvae at 5 dpf. Our goal was to find a concentration at which wildtype larvae do not respond to PTZ, and thus being sub-optimal in nature. We compared the potential sub-optimal concentrations with 1 µM PTZ, a seizure-inducing concentration. Table 1 demonstrates that exposure to 1µM PTZ causes hypermotility, indicated by increased small distance, large distance, total distance, and speed. Hypermotility is a reliable behavioral readout of seizure-like behavior in larvae [22,37]. We hypothesized that this sub-optimal concentration would elicit a heightened response in larvae with a mutation of *hcfc1a* relative to their wildtype siblings, rendering the allele more susceptible to seizure-like behavior. We monitored seizure-like behavior using ZebraBox technology after exposure to 1 µM, 10 pM, 1 pM, 0.1 pM, 0.01 pM, and 0.001 pM PTZ. In Figure 1A, we generated a radar plot to create a visual summary of the multiple parameters that were tracked using Viewpoint automated technology. These parameters included total distance, large distance, small distance, speed, large duration, small duration, large count, and small count. Seizure-like behavior is described as whirlpool-like behavior [38], with rapid movements that can be modeled by parameters such as increased fast distance (bursts), increased time spent in rapid movements (fast duration), and hyperlocomotion characterized by increased speed and total distance swam. As shown in Table 1, 1 µM, 10 pM, 1 pM, and 0.1 pM all induced a response after PTZ exposure, but to different degrees.

For example, seven unique parameters were increased after exposure to 1 µM PTZ. As we reduced the concentration of PTZ, we continued to observe considerable effects on the larvae, inducing behaviors indicative of seizure phenotypes such as hypermotility, increased movement, increased total distance, and increased time spent in small-duration movements. The number of parameters significantly affected by exposure was reduced as the dose of PTZ was reduced, except for 10 pM, which only led to the alteration of one parameter. In general, PTZ exposure increased movement and motility; however, at the 0.01 pM concentration, we observed reduced small count, small duration, small distance, large count, large duration, and total distance (Table 1). While it is counterintuitive that PTZ would elicit hypoactivity at any concentration, our observations at 0.01 pM are consistent with previous studies that have shown that low concentrations of PTZ can have opposite effects relative to higher concentrations [39]. At sub-convulsant doses in rats, PTZ has been shown to induce a conditioned place aversion [40,41], and a single sub-convulsive dose has been shown to induce an anxiety-like state [41]. Hypolocomotion is not sufficient to indicate anxiety in larval fish but can indicate a general depression of the central nervous system [35]. Zebrafish have immature circuits at this stage of development, and low-dose PTZ may cause disruptions that do not cause seizure-like behavior but do alter locomotion.

At the concentration of 0.001 pM, we observed no response to PTZ (Figure 1A,B). Based on this data, we concluded that a concentration of 0.001 pM of PTZ was not an effective seizure-inducing dose for wildtype animals at 5 dpf.

### 3.2. Mutation of hcfc1a Does Not Increase Seizure Susceptibility to Low-Dose PTZ

**Figure 2:**
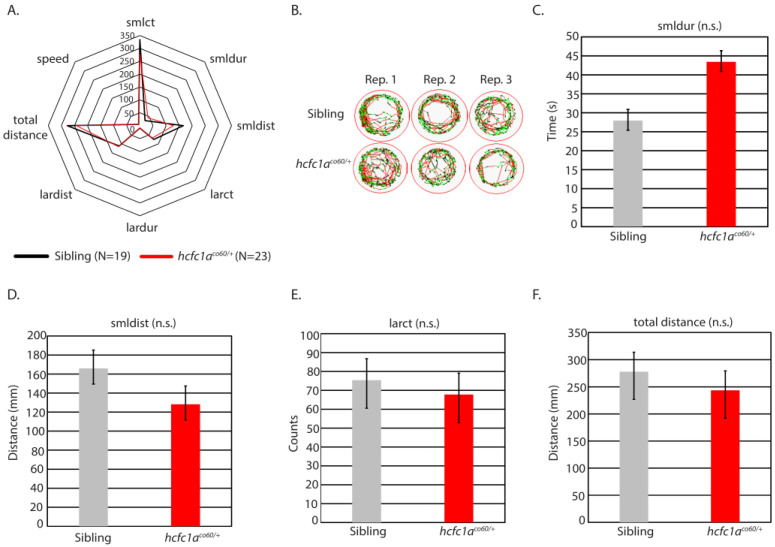
Mutation of *hcfc1a* does not increase response to PTZ. (**A**) Offspring of the *hcfc1a^co60/+^* allele were treated with 0.001 pM PTZ and assessed for behavioral patterns using the ZebraBox technology. A radar plot was used to compare the behavior of sibling wildtype (black) and heterozygous mutants (red). (**B**) Representative trace patterns were associated with behavior in (**A**). Three biological replicates were performed to achieve the total number of larvae, and panel (**B**) shows individual wells from each biological replicate. (**C**–**F**) Parameters from (**A**), for which there was a trending change in behavior, were individually analyzed using a bar graph and *t*-test. Small duration (**C**), small distance (**D**), large count (**E**), and total distance (**F**) are shown. Error bars represent the standard error of the mean. Sibling N = 19; *hcfc1a^co60/+^* N = 23. For representative trace patterns the color coding is consistent with the key in Figure 1.

The *hcfc1a^co60/+^* allele is a heterozygous viable, haploinsufficient nonsense mutation in the zebrafish *hcfc1a* gene, which has been characterized with abnormal neural development, behavioral deficits, and hyperactivated mTor [9,14]. *hcfc1a* is one conserved ortholog of *HCFC1* and, therefore, represents a putative system to study the function of HCFC1 in brain disease. Given that mutation of *hcfc1a* results in hyperactive mTor, which has been associated with seizure, we hypothesized that treatment of the *hcfc1a^co60/+^* allele with a sub-optimal concentration of PTZ would elicit a seizure-like response. We treated offspring of the *hcfc1a^co60/+^* allele with 0.001 pM PTZ (sub-optimal) and developed a radar plot to analyze all parameters (total distance, large distance, small distance, speed, large duration, small duration, large count, and small count). As shown in Figure 2A,B, wildtype siblings and heterozygous carriers did not demonstrate significant differences in behavior for all parameters examined after treatment with PTZ (0.001 pM). We analyzed heterozygous carriers because the allele is not homozygous viable. From the radar plot, we identified four parameters trending towards a significant response at sub-optimal doses of PTZ. For each of these parameters, we analyzed them independently. These included small duration (smldur), small distance (smldis), large count (larct), and total distance. As shown in Figure 2C, smldur trended towards increasing, but was not statistically significant. We did not observe any statistical change in smldist and larct (Figure 2D,E). Total distance was reduced (Figure 2F), but this change was not statistically significant, and thus, mutation of *hcfc1a* was not associated with an increased motility response at 0.001 pM PTZ.

### 3.3. Inhibition of mTor Reduces Seizure-like Behavior in Wildtype Animals

**Figure 3:**
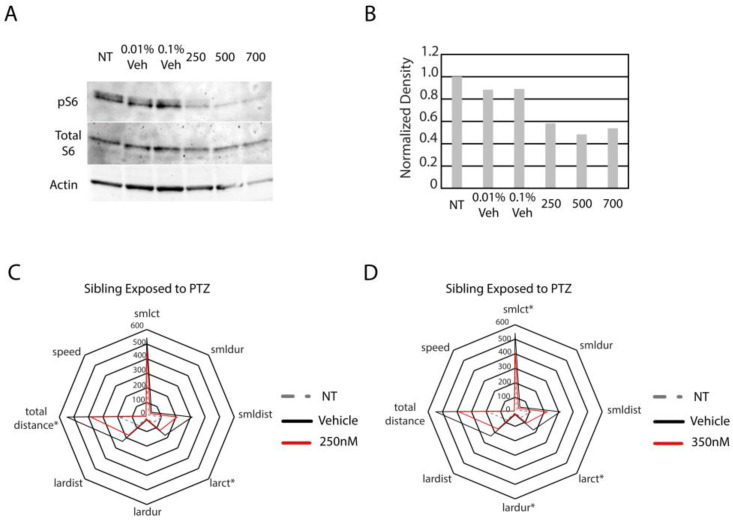
Optimization of torin1 treatment and PTZ response in wildtype animals treated with torin1. (**A**) Western blot with anti-phosphorylated ribosomal S6 protein, total S6 protein, and actin was performed on non-treated (NT), vehicle-treated (different percentages), and the indicated concentrations of torin1 (250 nM). For analysis in (**A**), 25 animals were harvested/group. (**B**) Quantification of (**A**). Subsequently, animals were treated with 250 nM (**C**) or 350 nM (**D**) torin1 and then exposed to 1 µM PTZ. For the analysis shown in (**C**), animal numbers are as follows: NT (N = 22), vehicle-treated (N = 20), and 250 nM (N = 19). Star plots were developed to analyze all parameters obtained directly from Zebralab software version 3.22. Asterisks indicate statistical significance (*p* < 0.05). For the analysis shown in (**D**), animal numbers are as follows: NT (N = 22), vehicle-treated (N = 20), and 350 (N = 18). For (**C**,**D**) * indicates *p* < 0.05.

We sought to determine whether hyperactive mTor in the *hcfc1a^co60/+^* allele causes increased reactivity to seizure-inducing concentrations of PTZ. We chose to treat larvae with 1 µM PTZ because our dose gradient demonstrated that 7/8 parameters increased after exposure (Table 1). Our first step was to inhibit mTor activity pharmacologically in wildtype larvae. We opted to inhibit mTor using torin1, a selective ATP-competitive inhibitor of mechanistic target of rapamycin complex 1 (mTorc1) and mechanistic target of rapamycin complex 2 (mTorc2). We performed a concentration gradient (100–700 nM) and monitored the level of phosphorylated S6 ribosomal protein (pS6) to determine the efficacy of the treatment and validate inhibition of mTor (Figure 3A,B and Figure S1). We observed no decrease in pS6 at 100 and 200 nM (Figure S1) but found decreased phosphorylation at concentrations equal to and higher than 250 nM. Thus, we treated wildtype larvae with 250 nM and 350 nM concentrations of torin1 and then exposed pretreated larvae to seizure-inducing concentrations of PTZ. As shown in Figure 3C and Table 2, treatment with 250 nM of torin1 reduced the overall activity of wildtype larvae and caused a significant reduction in total distance swam and the number of times the larvae were detected in large movements (large count). Treatment with 350 nM torin1 improved efficacy, resulting in a statistically significant reduction in large count, large duration (time spent in large movements), and small count (number of times the larvae are detected in small movements) (Figure 3D and Table 2). Total distance swam was also reduced, but was not statistically significant (*p* = 0.090). Thus, the increase in torin1 concentration was associated with a significant reduction in three parameters, whereas 250 nM reduced only two parameters. These data demonstrate that pretreatment with an mTor inhibitor reduced the response to PTZ in wildtype animals.

### 3.4. The Effects of mTor Inhibition on S6 Phosphorylation

**Figure 4:**
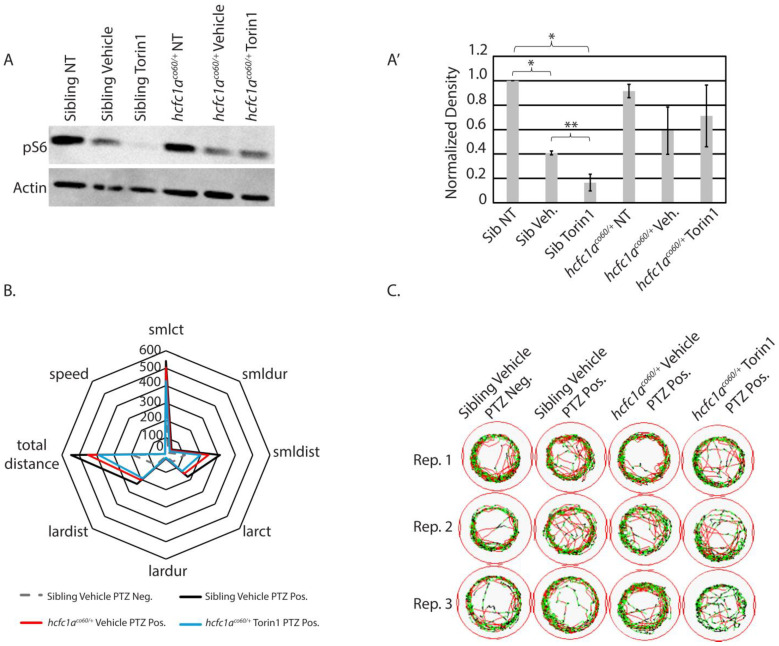
Effects of 250 nM torin1 treatment on pS6 and behavior. (**A**) Western blot was used to detect the phosphorylation of S6 ribosomal protein (pS6) in non-treated (NT), vehicle-treated (0.1% DMSO), or torin1-treated (250 nM) wildtype siblings or *hcfc1a^co/60/+^* larvae. Actin is used as a loading control, and an additional ponceau stain was performed. N = 14 larvae/group. (**A’**) Normalized density for Western blot detection, representing two biological replicates, was performed using Photoshop). The second Western blot is shown in Figure S2. Error bars represent the standard error of the mean. * *p* < 0.05, and ** *p* = 0.07. (**B**) Radar plot analyzing 8 parameters collected using ZebraBox technology in larvae treated with vehicle or torin1 and exposed to 1 µM PTZ (seizure-inducing). Comparison is provided for animals with no PTZ exposure (neg) and those with PTZ exposure (pos). (**C**) Sample trace patterns from the animals in (**B**). The trace patterns are examples from 3 independent replicates obtained from different parents on different days. Abbreviations: Rep. 1, 2, or 3 refers to biological replicate 1, 2, or 3. Colors in the trace patterns are equivalent to the key shown in Figure 1.

Next, we tested the effects of torin1 treatment on the *hcfc1a^co60/+^* allele. We hypothesized that a reduction in mTor signaling in mutant larvae would reduce the responsiveness to PTZ, given that mutation of *hcfc1a* results in hyperactive mTor signaling. To validate the effects of Torin1 in mutant animals, we performed a Western blot to detect pS6. Interestingly, treatment with vehicle control led to reduced levels of pS6 phosphorylation (Figure 4A). A biological replicate of this Western blot is shown in Figure S3, and the normalized density in Figure 4A’ includes densitometry from both replicates. However, torin1 is insoluble in water or ethanol, and its only indicated solubility on safety data sheets is DMSO. It has previously been used at 20 mg/kg in animal models and administered intraperitoneally. We validated that treatment of wildtype larvae with 0.1% DMSO has no effect on baseline behavior (Figure S2) relative to a non-treated control, and we assessed the effects of torin1 treatment on baseline behavior. The treatment of torin1 in the absence of PTZ increases activity, resulting in increased small count, total distance, large distance, and large duration (Figure S2). These data demonstrate an effect of torin1 that is not seen in DMSO control-treated animals. The effects of torin1 treatment without PTZ are also unique from its anti-epileptic effects in wildtype animals (Figure S2). However, because of the reduced pS6 in the DMSO group, we only compared behavior between the vehicle control groups and the torin1-treated groups. Torin1 severely abrogated pS6 in sibling wildtype, but torin1-treated mutants maintained a steady state level of pS6 relative to vehicle control-treated mutants (Figure 4A). These data substantiate dysregulation of mTor signaling in mutant animals.

### 3.5. Inhibition of mTor at 250 nM Reduces Small/Short Behavioral Parameters in Mutant Animals Exposed to Seizure-Inducing Doses of PTZ

**Figure 5:**
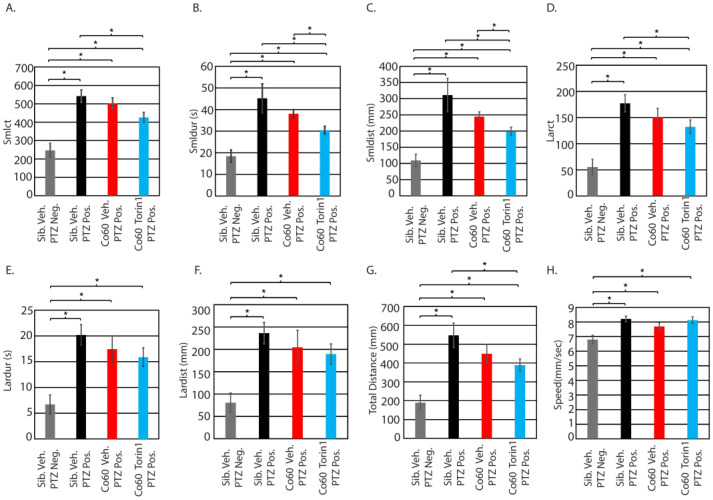
Inhibition of mTor reduces small-distance and small-duration movements. Parameters from Figure 4B were independently analyzed using ANOVA followed by a post hoc *t*-test between individual groups. Parameters include small count (**A**), small duration (**B**), small distance (**C**), large count (**D**), large duration (**E**), large distance (**F**), total distance (**G**), and speed (**H**). * *p* < 0.05. Comparison is provided for animals with no PTZ exposure (neg) and those with PTZ exposure (pos). All error bars represent the standard error of the mean. Sibling N = 22; Vehicle *hcfc1a^co60/+^* N = 28; torin1 *hcfc1a^co60/+^* N = 28.

We hypothesized that inhibition of mTor in mutant larvae would reduce responsiveness to PTZ. We began our analysis by confirming the effects of 1 µM PTZ on wildtype and mutant larvae at 5 dpf. Treatment with 1 µM PTZ induced substantial seizure-like phenotypes in sibling wildtype larvae treated with vehicle control (DMSO) (Figure 4B; black line relative to dashed gray line), as indicated by the fact that all parameters were significantly increased in wildtype siblings treated with vehicle control and 1 µM PTZ. A similar response was observed in *hcfc1a^co60/+^* larvae treated with PTZ (Figure 4B and Figure 5A–H). The mutant response to PTZ was reduced relative to wildtype, but there was not a statistically significant change in the response between mutants and wildtypes in any parameter tested (Figure 4B; black vs. red lines, and Figure 5A–H). Representative trace patterns are shown in Figure 4C. These include representative trace patterns from each group during each of the biological replicates. Colored detection in Figure 4C monitors speed according to the key in Figure 1B.

Next, we analyzed the response of torin1 (250 nM)-treated mutant larvae relative to vehicle control mutant larvae, both exposed to 1 µM PTZ (seizure-inducing). Initially, we detected a significant decrease in the response to PTZ in mutant larvae that were pretreated with torin1 (250 nM) relative to vehicle-treated sibling wildtype animals exposed to PTZ in the following parameters: smlct (Figure 5A), smldur (Figure 5B), smldist (Figure 5C), larct (Figure 5D), and total distance (Figure 5G). However, since vehicle-treated mutants show a slightly reduced response to 1 µM PTZ for all parameters relative to sibling vehicle exposed to PTZ (black bars vs. red bars), we decided the best comparison to determine the positive effects of torin1 treatment (250 nM) would be between vehicle control and torin1-treated (250 nM) mutants, both exposed to 1 µM PTZ. This approach allowed us to filter only the positive effects in mutant larvae. We detected a significant reduction in smldur and smldist between these groups (Figure 5B,C). These parameters are commonly associated with seizure-like behavior in zebrafish, as an increase in their values typically indicates heightened motility [35]. The observed decrease suggests that torin1 (250 nM) mitigated the smldur and smldist response. These effects mirrored the efficacy of torin1 in wildtype larvae.

### 3.6. Excessive Inhibition of mTor in the Context of hcfc1a Mutation Results in Refractory Epilepsy

**Figure 6:**
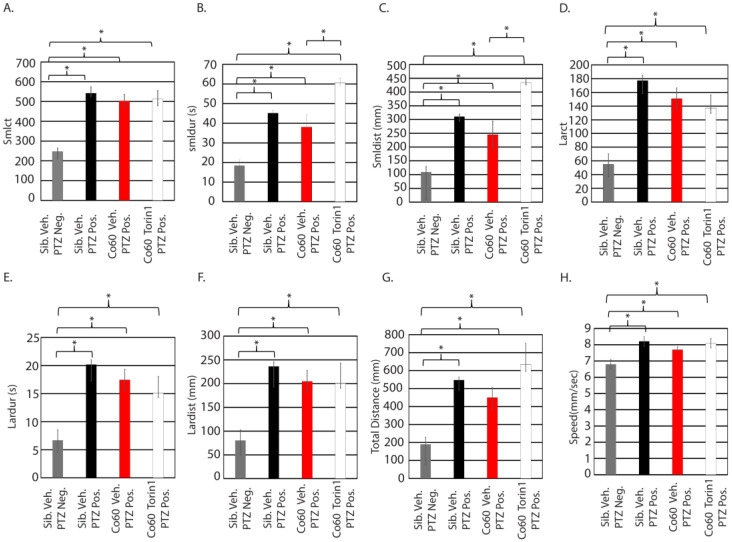
High dose inhibition of mTor increases the small distance and small duration in the *hcfc1a^co60/+^* allele. Zebrafish larvae were treated with 350 nM torin1 and exposed to 1 µM PTZ (seizure-inducing). Eight parameters were analyzed for each group: small count (**A**), small duration (**B**), small distance (**C**), large count (**D**), large duration (**E**), large distance (**F**), total distance (**G**), and speed (**H**). * *p* < 0.05. Numbers of animals per group are as follows: sib veh PTZ neg N = 20, sib Veh PTZ Pos N = 20, *hcfc1a^co60/+^* PTZ Pos N = 28, and *hcfc1a^co60/+^* torin1 PTZ Pos N = 30.

As shown in Figure 3D, pretreatment of wildtype larvae with 350 nM torin1 was more effective at reducing response to PTZ compared to 250 nM. Treatment with 350 nM reduced three parameters (lardur, larct, and smlct), whereas treatment with 250 nM only reduced two parameters (larct and total distance). Based on these data, we hypothesized that pretreatment of mutant larvae with 350 nM torin1 would be more efficacious than 250 nM torin1. We predicted that higher levels of torin1 would, consequently, reduce more parameters. Consistent with our previous results (Figure 5A–H), wildtype and mutant larvae responded to PTZ, with mutant larvae showing a mild reduction in response relative to wildtype siblings. The reduction was not statistically significant in any parameter. In contrast to pretreatment with 250 nM, mutant larvae treated with 350 nM torin1 had an exacerbated response to PTZ, characterized by increased smldur (Figure 6B) and smldist (Figure 6C) when compared to vehicle-treated mutant larvae also stimulated with PTZ. In each parameter, mutants treated with PTZ and 350 nM torin1 had a significant increase relative to sibling wildtype treated with vehicle control, indicating that the pretreatment of 350 nM torin1 was not effective at eliminating or alleviating the response to PTZ. Consequently, we conclude that pretreatment with 350 nM torin1 does not improve the response of mutant larvae in any parameter (Figure 6A,D–H) and increases the response to PTZ for the smldur and smldist parameters (Figure 6B,C). These data contrast with the observed effects in wildtype larvae (Figure 3D), in which treatment with 350 nM torin1 reduced activity in all parameters, with small count, large duration, and large count demonstrating statistical significance.

## 4. Discussion

Our study demonstrates that mutation of *hcfc1a* does not increase susceptibility to sub-optimal doses of PTZ. We found that 0.001 pM of PTZ does not produce seizure-like behaviors or hypermotility in wildtype or mutant larvae. Consequently, this dose is too low to be effective. However, higher concentrations of PTZ (1 µM) are effective in both wildtype and mutant larvae. The pharmacological inhibition of mTor signaling reduced the response to PTZ in zebrafish harboring a nonsense mutation in *hcfc1a*, but only in a dose-dependent manner (250 nM). While *hcfc1a* mutants did not exhibit increased response to sub-optimal PTZ doses, optimized mTor inhibition significantly attenuated small burst movements at seizure-inducing concentrations of PTZ. This attenuation was concentration-dependent, since higher concentrations of the mTor inhibitor, torin1, exacerbated the response to PTZ, suggesting that excessive manipulation of mTor in the context of *hcfc1a* mutation can lead to an increased response to PTZ. These findings align with an established role of mTor hyperactivation in epilepsy and underscore its partial contribution to seizure severity in *hcfc1a*-related pathology.

### 4.1. Integration with Existing Knowledge

The mTOR pathway is a well-documented regulator of synaptic plasticity, neuronal excitability, and epileptogenesis [15,42,43,44,45,46]. Our results extend this paradigm to *HCFC1*-associated disorders, where mTOR dysregulation may arise indirectly via upstream signaling defects, such as AKT hyperphosphorylation. Our previous studies have established that mutation of Hcfc1a leads to dysregulation of the PI3K/Akt/mTor axis. In these studies, the primary indicator of hyperactivated mTor is phosphorylation of S6, which would imply that mTorc1 is the primary mediator of hyperactivity. Treatment with rapamycin is sufficient to restore some phenotypes (radial glia), but not others (NPCs) [14]. At present, we do not know which cellular phenotypes underlie seizure development; consequently, we took a targeted approach to inhibit both mTor complexes. Future studies, with more targeted analysis of downstream mTor targets and independent inhibition of mTorc1 and mTorc2, are warranted.

Prior work linking *HCFC1* mutations to GABAergic deficits in mice [10] and radial glial abnormalities in zebrafish [9,14] suggest a multifaceted mechanism, wherein mTOR may underlie neurodevelopmental phenotypes and potentially intractable epilepsy in *cblX* syndrome and other HCFC1-related seizure disorders. We observed that mTOR inhibition partially mitigates seizure severity when provided at the appropriate dose, which parallels studies in tuberous sclerosis complex (TSC), where mTOR-targeted therapies reduce but do not eliminate seizures [15,16,17]. This partial efficacy highlights the likelihood of parallel pathways contributing to HCFC1-related epileptogenesis. Our results also reveal a dose-specific response, which may allude to the refractory nature of seizure phenotypes in *cblX* syndrome and may suggest additional pathways at play in *cblX* syndrome and related disorders. Our results warrant the testing of specific doses of mTOR inhibitors alongside traditional seizure treatments (i.e., valproic acid) in *cblX* and related HCFC1 disorders.

### 4.2. Mechanistic Implications

The blunted PTZ response in *hcfc1a* mutants, combined with persistent pS6 phosphorylation despite torin1 treatment (250 nM), suggests intrinsic mTOR pathway dysregulation. This resilience may stem from compensatory feedback mechanisms or crosstalk with other HCFC1-dependent pathways, such as THAP11/ZNF143-mediated transcription [1,2,8]. Additionally, the light-specific hypomotility reported in *hcfc1a* mutants [9] implies sensory-context-dependent phenotypes, emphasizing the need to evaluate seizures under diverse stimuli. The partial rescue of seizure severity by torin1 aligns with the role of mTOR in regulating neuronal excitability and glial function, both implicated in PTZ responses [45].

### 4.3. Limitations and Technical Considerations

For our study, we selected the zebrafish as a model system. Zebrafish larvae have been previously adapted as an idiopathic seizure model [22]. Specifically, PTZ has been shown to induce clonic-like convulsions in a dose-dependent manner that coincide with increased c-*fos* activity and extracellular readings from the fish optic tectum. There are currently over 325 studies in PubMed that study seizure-like phenotypes in zebrafish [37]. Zebrafish are now a respected model in the field and offer high-throughput analysis of potential therapeutic interventions. A total of 13 anti-epileptic drugs have been evaluated using the PTZ seizure paradigm. Most of the drugs (12/13) reduced locomotion, a readout for convulsive behavior, which was confirmed by open field testing. Overall, tests in zebrafish larvae with drug interventions mirror the data obtained in rodent models [23]. We did not perform electrophysiology recordings in our assay, and future studies that validate multiple mTor inhibitors should be supported by electrophysiology or transgenic calcium recordings. However, based on the effects of mTOR inhibition in mice, we anticipate that zebrafish will show similar consistencies.

Unexpectedly, vehicle (DMSO) treatment reduced pS6 levels in wildtype larvae, complicating direct comparisons between untreated and torin1 groups. This underscores the importance of vehicle controls in pharmacological studies. However, the molecular changes did not affect baseline behavior (Figure S2). Furthermore, our focus on PTZ, a GABA receptor antagonist, may overlook seizure mechanisms specific to other neurotransmitter systems. Future studies using alternative proconvulsants (e.g., kainic acid) could clarify whether mTOR’s role is stimulus-dependent. Additionally, the use of larval zebrafish, while advantageous for high-throughput screening, limits the investigation of chronic epilepsy models or cognitive comorbidities.

### 4.4. Therapeutic Relevance

Our findings support precise dose-dependent mTOR inhibition as a potential therapeutic strategy for mitigating seizure severity in *cblX* syndrome. However, the incomplete rescue by torin1 suggests that combinatorial therapies targeting both mTOR and ancillary pathways (e.g., GABAergic signaling [10,11] or epigenetic regulators like ASXL1 [47]) may be necessary. Clinical translation will require careful evaluation of mTOR inhibitors in HCFC1 patient-derived models, given the pathway’s pleiotropic roles in neurodevelopment.

### 4.5. Future Directions

Building on these findings, several avenues merit exploration to deepen our understanding of HCFC1-related epileptogenesis and refine therapeutic strategies. First, the mechanistic dissection of the interplay between HCFC1, mTOR, and downstream effectors could clarify how transcriptional dysregulation converges on neuronal hyperexcitability. Integrating multi-omics approaches such as single-cell RNA sequencing and phosphoproteomics in *hcfc1a* mutants may identify novel mTOR-independent pathways contributing to seizure phenotypes. For instance, the interaction between HCFC1 and epigenetic regulators like ASXL1 [48] and transcriptional complexes such as THAP11/ZNF143 [1] warrants investigation, as these interactions could modulate synaptic gene networks or glial–neuronal crosstalk. Parallel studies in patient-derived induced pluripotent stem cell (iPSC) models would further bridge zebrafish findings to human pathophysiology.

Second, expanding seizure paradigms beyond PTZ could uncover stimulus-specific mechanisms. Optogenetic induction of seizures or audiogenic stimuli may reveal sensory-modulated epileptogenesis, particularly given the light-dependent hypomotility observed in *hcfc1a* mutants [9]. Additionally, chronic seizure models in adult zebrafish could assess whether mTOR inhibition alters epilepsy progression or comorbid cognitive deficits, which are hallmarks of *cblX* syndrome. Third, longitudinal studies evaluating mTOR inhibitor safety, dosage, and efficacy across developmental stages are critical. While acute dose-dependent torin1 treatment reduced seizure severity in larvae, prolonged inhibition during neurodevelopment at larger doses might exacerbate HCFC1-related deficits, such as radial glial migration [49]. Dose- and timing-dependent studies could optimize therapeutic windows while minimizing off-target effects.

Finally, combinatorial therapies targeting mTOR and complementary pathways such as GABAergic signaling [10] or ASXL1-mediated AKT activation [9] should be explored. Given the partial rescue by torin1, synergistic drug screens could identify compounds that enhance mTOR inhibition or bypass HCFC1-dependent metabolic defects. Collaborations with clinical researchers to profile mTOR activity in *cblX* patients would validate zebrafish findings and accelerate translational applications. Together, these efforts promise to unravel the complexity of HCFC1-associated epilepsy and advance personalized treatment paradigms for neurodevelopmental disorders linked to transcriptional co-factor dysfunction.

## 5. Conclusions

In summary, our work positions mTOR as one potential modulator of seizure severity in *hcfc1a*-deficient zebrafish, offering a mechanistic bridge between HCFC1 dysfunction and epilepsy. While therapeutic targeting of mTOR holds promise, the partial rescue observed here underscores the complexity of *cblX* pathophysiology and the need for multifaceted treatment strategies.

## Figures and Tables

**Table 1:** Empirical derivation of sub-optimal concentrations of PTZ and the parameters monitored.

PTZ	Small Count	Small Duration	Small Distance	Large Count	Large Duration	Large Distance	Total Distance (mm)	Speed	# of Parameters Affected
1.0 µM	+59.17	NC	+33.32	+39.7	+5.42	+66.32	+99.62	+0.67	7
10 pM	NC	NC	NC	NC	NC	NC	NC	+0.83	1
1 pM	+194.93	+18.73	+116.85	NC	NC	NC	+108.63	−1.56	5
0.1 pM	+51.26	NC	NC	+19.70	+2.55	NC	NC	NC	3
0.01 pM	−49.65	−4.92	−32.97	−22.48	−2.62	NC	−59.34	NC	6
0.001 pM	NC	NC	NC	NC	NC	NC	NC	NC	NC

NC: No significant change was identified. The alternating shades of gray between rows indicate the different concentration tested. The values provided in the Table indicate the increased (+) or decreased (−) response relative to untreated control larvae. For each condition, 3 biological replicates were performed to obtain a total N of 48. In the final column # is used to abbreviate the total number of parameters affected.

**Table 2:** Effectiveness of torin1 treatment on wildtype larvae.

Torin1	Small Count	Small Duration	Small Distance	Large Count	Large Duration	Large Distance	Total Distance (mm)	Speed	# of Parameters Affected
250 nM	NC	NC	NC	−48.41	NC	NC	−163.30	NC	2
350 nM	−127.81	NC	NC	−67.52	−7.35	NC	NC	NC	3

NC: No significant change was identified. The values provided in the Table indicate the increased (+) or decreased (−) response relative to larvae treated with vehicle control and pentylenetetrazol. The alternating shades of gray and white between rows indicate the different concentration tested. In the final column # is used to abbreviate the total number of parameters affected.

## Data Availability

All data and reagents are available upon request from the corresponding author.

## Supplementary Materials

The following supporting information can be downloaded at https://www.mdpi.com/article/10.3390/jdb14010002/s1: Supplemental Figure S1: Western blot analysis of torin1 inhibition. Supplemental Figure S2: Radar plot summarizing behavioral analysis of vehicle control and torin treated wildtype larvae. Supplemental Figure S3: Biological replicate for Western blot shown in Figure 4.
