# Supplementary material for: A Zebrafish Seizure Model of cblX Syndrome Reveals a Dose-Dependent Response to mTor Inhibition
Source: J Dev Biol. 2025 Dec 25;14(1):2. doi: 10.3390/jdb14010002 (PMC12821537; doi:10.3390/jdb14010002)
Supplement: Supplementary file 1 [file jdb-14-00002-s001.zip › jdb-3998061-supplementary.pdf]

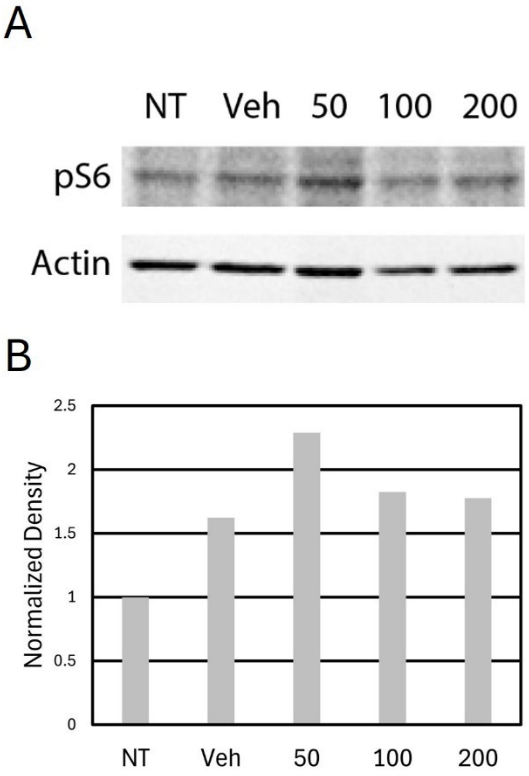

Supplemental Figure S1: (A) Western blot was to determine the optimal concentration of torin1 to reduce mTor activity. Larvae were treated as described in the materials and methods and antibodies to detect phosphorylated S6 ribosomal protein and actin were used. NT is non-treated control; veh is vehicle control (DMSO). N=25/group (B) Normalized density of (A).

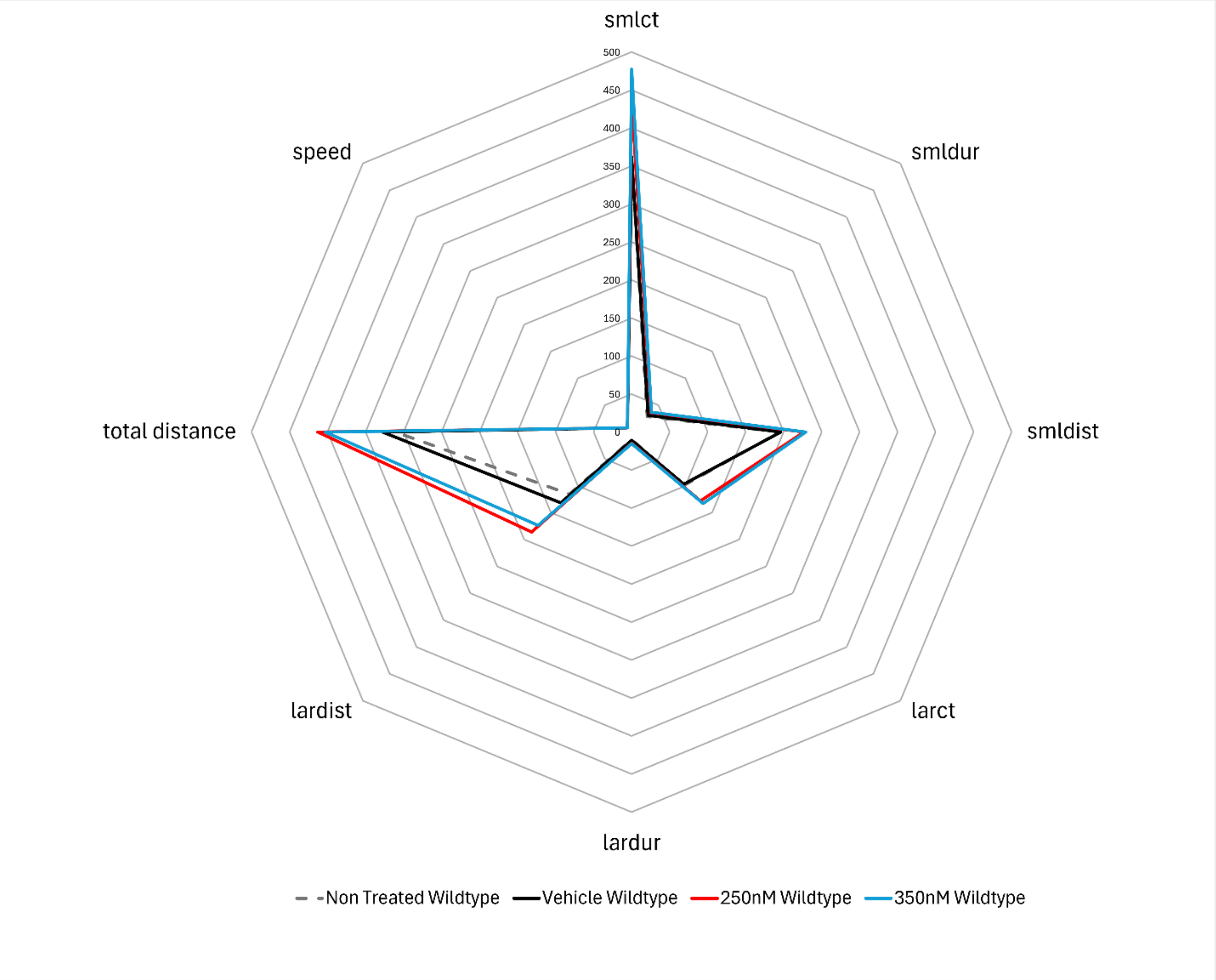

Supplemental Figure S2: Radar plot analyzing 8 unique behavioral patterns in larvae at 5 days post fertilization. Analysis compares non treated wildtype, vehicle treated (0.01% DMSO), 250nM torin1 treated, and 350nM torin1 treated larvae. Vehicle control treated animals do not demonstrate any significant changes in behavior relative to non-treated controls. The addition of 250 or 350nM torin1 reduces pS6 and leads to increased motility in the absence of PTZ or any other stimulate. These analyses were performed as part of the global analysis. They indicate internal controls prior to the exposure to PTZ and as indicated in materials and methods represent baseline control measurements.

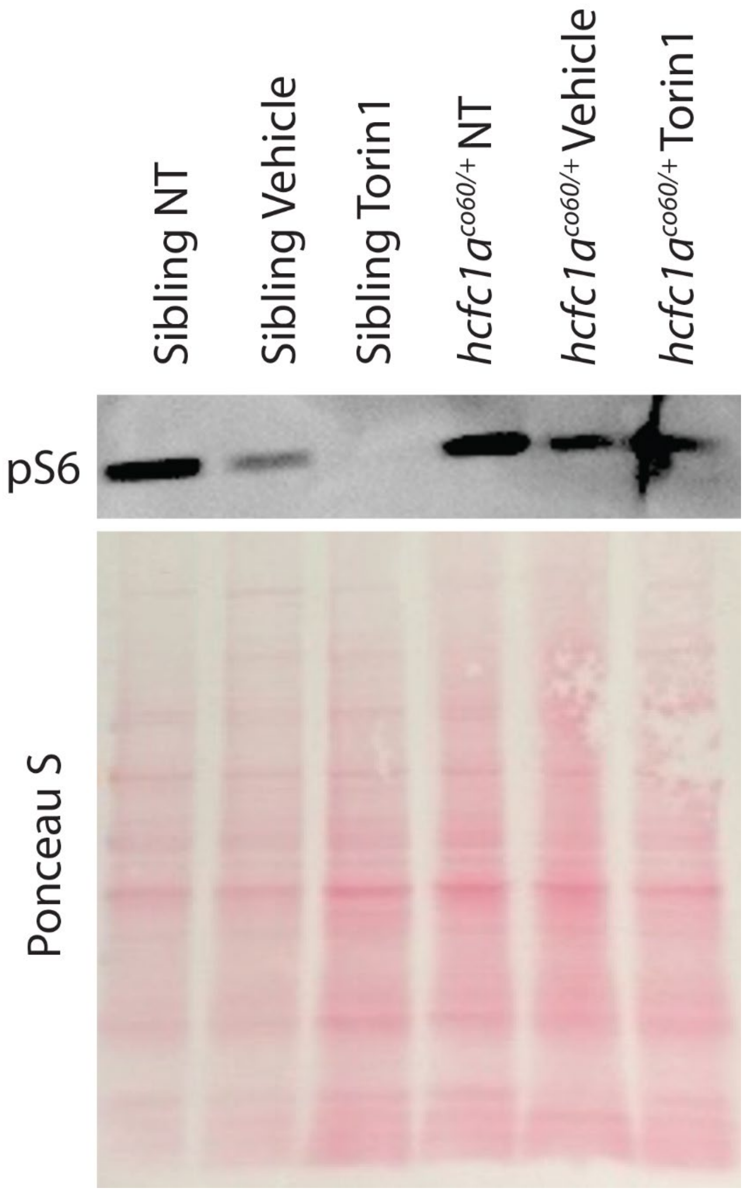

Supplemental Figure S3: Biological replicate for data represented in Figure 4, which validates the phosphorylation of ribosomal protein S6 (pS6). Total protein was isolated from a pool of larvae at 5 days post fertilization post treatment with vehicle control (veh) or torin1. Vehicle and treatment are presented in sibling (sib) wildtype or heterozygous carriers of the *hcfc1a*$^{co60/+}$ allele. A non-treatment control for each genotype is shown (NT). Ponceau S was used as a loading control and normalized density of both biological replicates was performed in Figure 4.
